# Simultaneous MEG and EEG source imaging of electrophysiological activity in response to acute transcranial photobiomodulation

**DOI:** 10.3389/fnins.2024.1368172

**Published:** 2024-05-16

**Authors:** Tyrell Pruitt, Elizabeth M. Davenport, Amy L. Proskovec, Joseph A. Maldjian, Hanli Liu

**Affiliations:** ^1^Department of Radiology, UT Southwestern Medical Center, Dallas, TX, United States; ^2^Department of Bioengineering, University of Texas at Arlington, Arlington, TX, United States

**Keywords:** magnetoencephalography (MEG), transcranial photobiomodulation (tPBM), non-invasive neuromodulation, source localization, electrophysiological responses

## Abstract

**Introduction:**

Transcranial photobiomodulation (tPBM) is a non-invasive neuromodulation technique that improves human cognition. The effects of tPBM of the right forehead on neurophysiological activity have been previously investigated using EEG in sensor space. However, the spatial resolution of these studies is limited. Magnetoencephalography (MEG) is known to facilitate a higher spatial resolution of brain source images. This study aimed to image post-tPBM effects in brain space based on both MEG and EEG measurements across the entire human brain.

**Methods:**

MEG and EEG scans were concurrently acquired for 6 min before and after 8-min of tPBM delivered using a 1,064-nm laser on the right forehead of 25 healthy participants. Group-level changes in both the MEG and EEG power spectral density with respect to the baseline (pre-tPBM) were quantified and averaged within each frequency band in the sensor space. Constrained modeling was used to generate MEG and EEG source images of post-tPBM, followed by cluster-based permutation analysis for family wise error correction (*p* < 0.05).

**Results:**

The 8-min tPBM enabled significant increases in alpha (8–12 Hz) and beta (13–30 Hz) powers across multiple cortical regions, as confirmed by MEG and EEG source images. Moreover, tPBM-enhanced oscillations in the beta band were located not only near the stimulation site but also in remote cerebral regions, including the frontal, parietal, and occipital regions, particularly on the ipsilateral side.

**Discussion:**

MEG and EEG results shown in this study demonstrated that tPBM modulates neurophysiological activity locally and in distant cortical areas. The EEG topographies reported in this study were consistent with previous observations. This study is the first to present MEG and EEG evidence of the electrophysiological effects of tPBM in the brain space, supporting the potential utility of tPBM in treating neurological diseases through the modulation of brain oscillations.

## 1 Introduction

Transcranial magnetic stimulation (TMS) and transcranial direct current stimulation (tDCS) are well-known noninvasive methods for modulating neuronal activity in the human brain. They stimulate neural activity and alter electrophysiological signals; thus, they have been explored as therapeutic tools. A third method of noninvasive neuromodulation, known as transcranial photobiomodulation (tPBM), offers another approach for noninvasive modulation of neural activity (Wang et al., 2017; Hamblin and Huang, 2019; Gonzalez-Lima, 2021). This involves transcranial exposure of the human head to near-infrared (NIR) light, which can penetrate the scalp and skull and reach the brain. Commonly used wavelengths in tPBM include 660 nm, 800–850 nm, and 1,064–1,070 nm, which activate cellular mechanisms and promote ATP production (Fear et al., 2023) and local blood oxygenation (Wang et al., 2017; Baik et al., 2021). Many studies offer substantial evidence that tPBM is effective to enhance human cognition and brain function in both healthy adults (Hamblin and Huang, 2019; Gonzalez-Lima, 2021; Qu et al., 2022; Zhao et al., 2022) and in a variety of brain disorders (Wang et al., 2017; Hamblin and Huang, 2019; Gonzalez-Lima, 2021; Nizamutdinov et al., 2022). Recent efforts have also been made to explore tPBM as a method to treat Alzheimer’s disease (AD; Hamblin, 2019; Baik et al., 2021; Chan et al., 2021; Hamblin and Salehpour, 2021; Nizamutdinov et al., 2021).

Numerous studies have reported that targeted tPBM leads to performance improvements in prefrontal cortex-related tasks, underscoring the potential benefits of this approach for mental health interventions (Chao, 2019; Salehpour et al., 2019; Spera et al., 2021). In particular, Gonzalez-Lima et al. showed that right-prefrontal tPBM enhances neural efficiency and outcomes in cognitive tasks (Barrett and Gonzalez-Lima, 2013; Gonzalez-Lima, 2021). This body of work provides us with a compelling rationale for selecting the right prefrontal cortex as the tPBM delivery site for this study.

Given the potential of tPBM as an intervention, it is necessary to better understand its mechanism of action to achieve effective treatment outcomes. The well-accepted, evidence-supported theory is that cytochrome C oxidase within the cellular mitochondria of neurons absorbs NIR light, releases nitric oxide, and upregulates ATP production (Wang et al., 2016; Pruitt et al., 2020; Fear et al., 2023). However, only a few studies have reported tPBM-induced effects on electrophysiological activity in the human brain, recorded using electroencephalography (EEG; Zomorrodi et al., 2019; Ghaderi et al., 2021; Wang et al., 2021; Shetty et al., 2023). EEG measures electrical potentials on the scalp surface and has been widely used in research and clinical settings to monitor neural and electrophysiological activity. Several recent EEG-based studies by our group have demonstrated that continuous tPBM increases alpha (8–13 Hz) and beta (13–30 Hz) power near the tPBM site (Wang et al., 2019, 2021) and enhances brain connectivity from the stimulation site to different remote cortical regions (Shahdadian et al., 2022; Wang et al., 2022). However, EEG responses to tPBM to date have limited spatial resolution and suffer from the typical electrical conductance issues for accurate brain source localization.

Magnetoencephalography (MEG) is an electrophysiological imaging method that measures human brain activity by recording extracranial magnetic signals instead of direct electrical currents at the scalp (Bagic and Sata, 2007; Singh, 2014). Compared with EEG (64-channel electrodes), MEG can provide more accurate electrophysiological information and better spatial resolution for source localization. Typical MEG scanners have approximately 300 sensors for signal detection and, because the magnetic fields are unperturbed by the various tissues, simpler head models can be employed when reconstructing the source images. In addition, MEG requires a much easier preparation for helmet placement without any concern for electrical impedance. Thus, MEG is a valuable neuroimaging tool for investigating the electrophysiological activity in response to tPBM in the human brain.

Our intention of this study was not to compare MEG and EEG modalities to determine which one is superior or which one provides better insights. Instead, our objective was to utilize the complementary strengths of both modalities to gain and confirm the consistent effects of tPBM on the brain. EEG, with a rich history in its research, serves as a foundational tool and offers excellent insight into the brain’s response to tPBM, which can be compared to previous studies. In contrast, MEG complements EEG with a superior spatial resolution and is particularly effective in localizing superficial brain activity. Since no study on tPBM-induced MEG effects and very limited studies on tPBM-induced EEG effects can be found in the literature, this study focused on utilizing the strengths of each modality to reveal tPBM’s effect on the brain and investigated their agreements. We plan to leverage the complementary strengths of both MEG and EEG to gain a more comprehensive understanding of the effects of tPBM on the brain in the near future, because of the key benefits of combining the strengths of both modalities (Puce and Hamalainen, 2017).

Here we present novel concurrent MEG and EEG measurements obtained from 25 healthy participants in response to the acute 1,064-nm laser tPBM delivered to the right forehead. This study provides source-space mapping and comparisons of participants’ electrophysiological responses obtained using MEG and EEG (MEG/EEG). To the best of our knowledge, this is the first study to employ concurrent MEG/EEG measurements and a constrained source modeling method to quantify and image the significant electrophysiological effects of 1,064-nm laser tPBM in the cortical source space. Specifically, the novel findings and characteristics include the following: (1) tPBM-induced increases in normalized MEG/EEG spectral powers consistently in two frequency bands (i.e., alpha and beta bands); (2) tPBM enabled significant increases in MEG/EEG power in local and remote brain regions; and (3) MEG/EEG-derived observations in this study were consistent with those in previous EEG findings.

## 2 Materials and methods

### 2.1 Participants

A total of 25 healthy young participants (13 female and 12 male; mean age ± s.d. = 25.6 ± 4.8 years) were recruited from the University of Texas Southwestern Medical Center (UTSW) and surrounding areas for this study. Participants who met the following inclusion criteria were enrolled in the study: (1) no prior diagnosis of a psychiatric disorder, (2) no history of brain injury or nervous disorders, (3) no consumption of caffeine or nicotine within 2 h prior to the experiment, and (4) no current pregnancy. This study was conducted in accordance with the ethical principles outlined in the Declaration of Helsinki (World Medical Association, 2013). The study protocol was approved by the Institutional Review Board (IRB) of UTSW. All participants provided written informed consent prior to participating in the study, ensuring their full understanding and agreement to participate.

### 2.2 Instrumentation

The tPBM protocol utilized a continuous-wave 1,064-nm laser system (CG-5000, Cell Gen Therapeutics, LLC; Dallas, TX, United States). This light stimulation device has received FDA clearance for the treatment of inflammation and pain relief. The laser unit delivered a power density of 250 mW/cm^2^ across a 4.2-cm-diameter aperture during stimulation of the right prefrontal cortex of the participants, as reported in our earlier studies (Wang et al., 2019, 2021).

**Figure 1:**
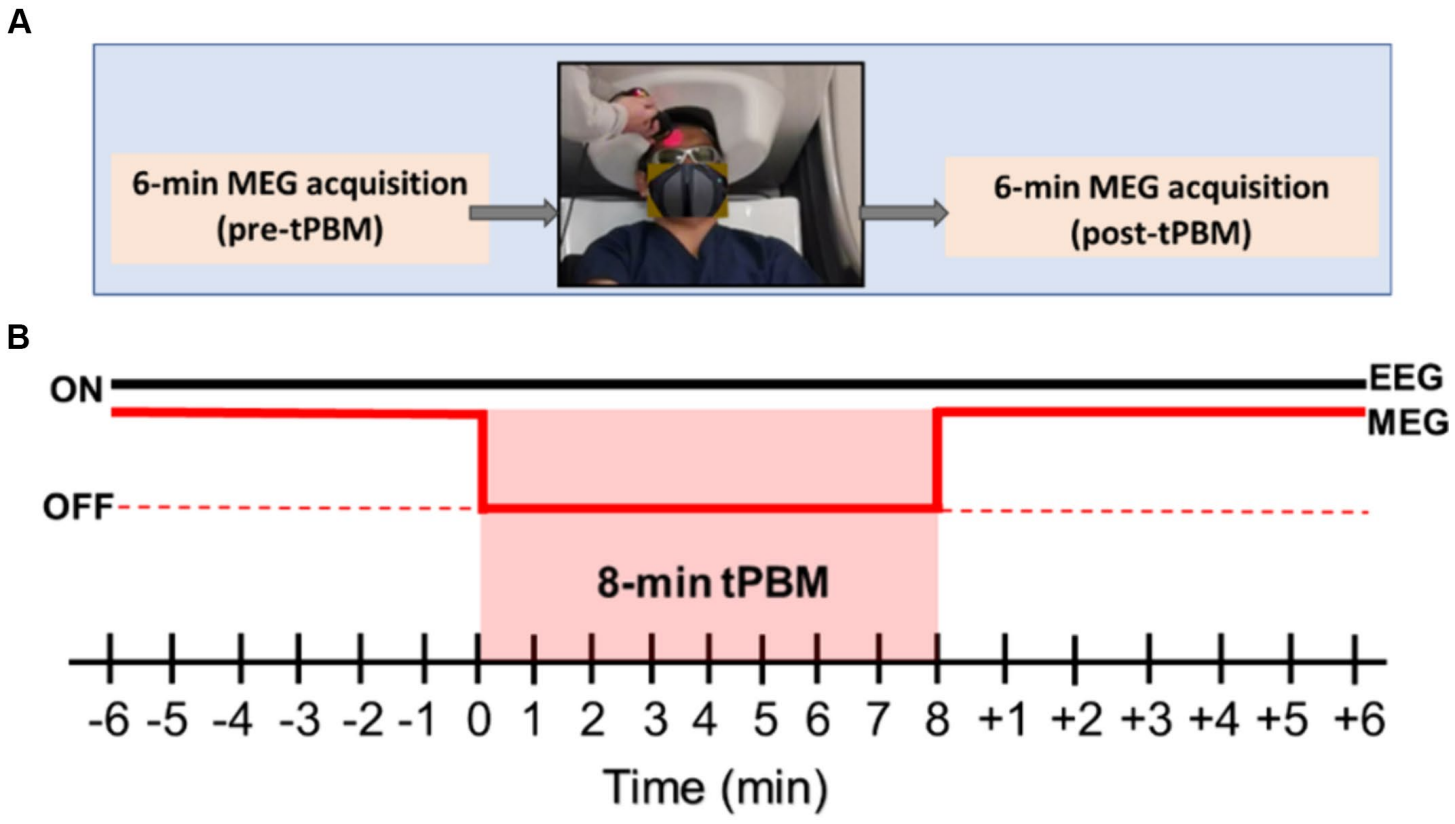
**(A)** The overall experimental protocol for MEG data acquisition includes 6-min pre-tPBM MEG acquisition, 8-min tPBM on the right forehead of a participant delivered outside of the MEG helmet, followed by another 6-min MEG data collection. **(B)** Detailed MEG and EEG acquisition sequences for all three pre-, during, and post-tPBM phases for 20 min. Specifically, EEG data were continuously collected in 20 min, while MEG data were acquired only during the 6-min pre-and post-tPBM periods.

The MEG data were acquired using a MEGIN Neuromag TRIUX Neo system, which included 204 planar gradiometers and 102 magnetometers, with a sampling rate of 1 kHz during the 6-min pre- and post-tPBM epochs. Because of the large size of the MEG helmet and the close distance to the human head required for MEG scans, it was impossible to deliver laser light concurrently with the MEG data acquisition. During the 8-min tPBM stimulation, the participant was asked to sit in a lower position with the forehead exposed below the MEG helmet for light delivery (see the photo in Figure 1A).

Structural T1-weighted brain MR images for each participant were acquired during the visit using a 3 T Siemens scanner and an MPRAGE sequence with a resolution of 0.9 mm (TR = 1,900 ms; TE = 2.93 ms; TI = 900 ms; flip angle = 9°; 176 slices). These T1 images were used for source-space localization of the MEG/EEG measurements. The MRI data were normalized to the Montreal Neurological Imaging (MNI) space using SPM12 (Tadel et al., 2011).

### 2.3 Experiments

Participants were first seated comfortably, followed by the placement of an EEG cap on their heads. The EEG electrodes were cleaned with an alcohol solution and then covered with a conductive EEG electrolyte-rich gel. The impedance of each electrode was checked to ensure they were under 5 kΩ. Next, Head Position Indicator (HPI) coils were inserted into the EEG cap slots. The HPI coils allowed tracking and continuous position and orientation monitoring of the head in the MEG machine during the experiment. Anatomical fiducials were marked and digitized using the Polhemus system, which also marked the EEG electrodes, HPI coil locations, and an additional 300 scalp points for co-registration. Next, the participants were escorted into the MEG room and positioned in the MEG helmet. As shown in Figure 1B, a pre-tPBM resting eyes-open epoch of 6 min was recorded using simultaneous MEG/EEG. After this period, the participants were lowered to expose the tPBM stimulation site (1 cm below Fp2 in the right frontal region), and protective goggles (900–1,000 nm,5+, 1,000–2,400 nm,7+; 2,900–10,600 nm,7+) were worn by both participants and the research staff in the room. EEG data were continuously collected prior to, during, and after tPBM (see Figure 1B). The participants received active tPBM at 250 mW/cm^2^ light irradiance for 8 min. After tPBM stimulation, the participants were raised back into the MEG helmet and a 6-min post-tPBM resting eyes-open epoch was recorded using simultaneous MEG/EEG. Finally, the participants were led to an MRI scanner for T1-weighted MRI scans, which were utilized to co-register with the anatomical fiducials marked for MEG sensors.

### 2.4 Data processing for MEG (EEG) data in sensor space

**Figure 2:**
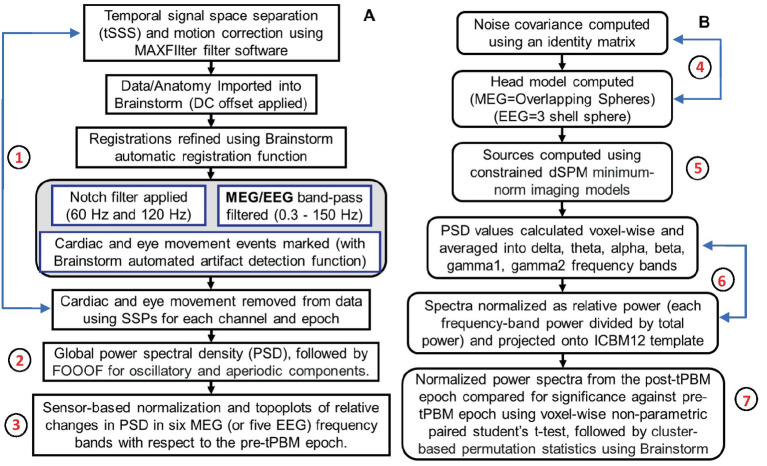
Two flowcharts outlining data processing steps in **(A)** sensor space and **(B)** source space. Specifically, steps 1–3 include data pre-processing, noise removal, global PSD calculations, and MEG/EEG power topoplots in sensor space. Steps 4–7 list procedures used to image significant tPBM effects in brain source space. These steps were performed for both MEG and EEG data processing except that they included different frequency bandwidths and used different head models for brain source imaging.

Several data processing steps were performed to reveal significant tPBM effects in sensor space, as outlined in Figure 2A.

#### 2.4.1 Pre-processing (step 1)

The acquired MEG data were processed for temporal signal space separation (tSSS) and motion correction using MEGIN MAXFilter software, followed by importing the data into Brainstorm (Tadel et al., 2011). Registrations were refined using automatic registration functions in Brainstorm. Next, notch filters at 60 and 120 Hz were applied to eliminate electrical line harmonics, followed by a bandpass filter of 0.5–150 Hz to select the frequencies of interest. Cardiac noise and eye movements were marked in the data using Brainstorm’s artifact detection function and then removed using Source Space Projectors (SSPs) for each channel and epoch. These steps were repeated for EEG data collected concurrently in the pre-and post-tPBM epochs.

#### 2.4.2 Calculation of global power spectrum density (step 2)

After data pre-processing, the power spectrum density (PSD) of each MEG (or EEG) channel was calculated using the Welch method (Welch, 1967; Percival and Walden, 1993; Stoica and Moses, 2005; Haykin and Van Veen, 2007; Bendat and Piersol, 2011). Each PSD curve of the MEG (or EEG) signal was quantified in mV^2^/Hz as a function of frequency, *f*, which expresses spectral bands between 0.5–150 Hz for MEG (or 0.5–80 Hz for EEG). Global PSDs were calculated by averaging all 204 planar gradiometers for MEG and all EEG channels for both pre-and post-tPBM epochs.

Next, we utilized the Fitting Oscillations and One-Over-F (FOOOF) algorithm to accurately attribute MEG (or EEG) PSD changes to oscillatory activity. FOOOF is a sophisticated analysis technique specifically designed to segregate a signal into oscillatory and aperiodic components. This algorithm enabled us to isolate the oscillatory changes induced by tPBM accurately, distinct from the aperiodic activity present in the MEG/EEG data. This marked a significant advancement in our analysis, allowing for a more nuanced interpretation of electrophysiological alterations following tPBM intervention.

#### 2.4.3 Normalization of channel-wise PSD (step 3)

To quantify tPBM-induced changes in MEG (or EEG) band-averaged power with respective to the pre-tPBM condition, we utilized eq. (1) to normalize channel-wise power changes in each frequency band relative to those at the pre-stimulation condition. In other words, eq. (1) calculates a percent change in frequency-band-averaged MEG (or EEG) power induced by tPBM for each sensor.


(1) $$\begin{array}{c} MEG\;\left(EEG\right)power\\ change\left(i,f\right) \end{array}=\frac{\begin{array}{l} averagedpower_{post}\left(i,f\right)-\\ averagedpower_{pre}\left(i,f\right) \end{array}}{averagedpower_{pre}\left(i,f\right)}100\%$$

where *i* indicates the number of sensors and *f* represents the frequency band. For MEG, *i* = 1, 2, … 204, and *f* = 6 for delta (1–3 Hz), theta (4–7 Hz), alpha (8–12 Hz), beta (13–30 Hz), gamma1 (31–80 Hz), and gamma2 (81–150 Hz). For EEG, *i* = 1, 2, … 64, and *f* = 5 for delta (1–3 Hz), theta (4–7 Hz), alpha (8–12 Hz), beta (13–30 Hz), and gamma1 (31–80 Hz). High Gamma is not included in the EEG data pipeline due to the consideration of increased prevalence of noise from artifacts in this range and to match with our previous works.

### 2.5 Data processing for MEG (EEG) data in source space

Each T1-weighted MRI scan from each participant was co-registered with anatomical fiducials of the MEG/EEG sensors to ensure spatial alignment between the different modalities. Next, the MNI-ICBM152 template source grid, a widely recognized brain atlas, was warped to fit each participant’s individual T1 MRI scan. This process enabled the creation of personalized head models, onto which MEG (EEG) power changes were projected for source space analysis. Standardization of the source grids across participants facilitated the identification of consistent patterns across different participants. Subsequently, four processing steps were performed to reveal the significant tPBM effects in the source space, as outlined in Figure 2B.

#### 2.5.1 Forward head model building for source imaging (step 4)

For the source space analysis, MEG gradiometer readings were used for MEG source identification because of their superior localization ability and resistance to noise artifacts compared with magnetometer data (Hämäläinen et al., 1993; Vrba and Robinson, 2001; Hillebrand and Barnes, 2002; Taulu et al., 2004; Taulu and Simola, 2006; Gross et al., 2013). As shown in Figure 2B, the noise covariance was computed using an identity matrix with the default assumption that the noise in the MEG channels was uncorrelated across sensors and time points, and only the diagonal elements of the covariance matrix were considered. Next, the cortex surface head models were computed using overlapping spheres for MEG data (and a 3-shell sphere for EEG data).

#### 2.5.2 Inverse modeling for source imaging (step 5)

In neuromagnetism, combining MEG signals and MRI data through constrained modeling (CM) in Brainstorm, specifically using the dSPM technique, effectively localizes neural activity sources. This approach, frequently used in clinical settings for its localization accuracy, was applied in our study to examine cortical responses to tPBM, using constrained dSPM minimum-norm imaging models (Hämäläinen and Ilmoniemi, 1994; Dale et al., 2000; Gross et al., 2001; Sekihara et al., 2005; Lin et al., 2006; Tadel et al., 2011).

#### 2.5.3 Calculation of voxel-wise changes in spectral power induced by tPBM (step 6)

After obtaining the cortex-constrained sources, the voxel-wise (306,716 Vertices, 613,424 Faces) time series were transformed into frequency-domain PSD values using the Welch method and averaged into six frequency bands: delta, theta, alpha, beta, gamma1, and gamma2. Subsequently, the voxel-wise spectral power in each band was normalized with respect to the total power (0.5–150 Hz) and projected onto the MNI-ICBM152 template model for data visualization and further processing using a common anatomical framework. This normalization step was performed separately for both pre-and post-tPBM, followed by subtraction of the pre-tPBM spectral powers from the respective post-tPBM powers.

#### 2.5.4 Statistical analysis of source space images (step 7)

Steps 1 to 6 were repeated for all participants to obtain the tPBM-induced PSD changes in source space for the group-level statistical analysis. Next, we employed cluster-based permutation statistics, as implemented in Brainstorm, to account for family wise error corrections on the PSD-projected cortex (grid) space across different frequency bands (Maris and Oostenveld, 2007). This cluster-wise analysis enabled us to identify the significant effects of tPBM on the cortical regions of the human brain. A corrected significance of a corrected significance level of 0.05 was used to produce frequency-specific source-space t-value topographies. Steps 6–8 were repeated for EEG data analysis.

## 3 Results

### 3.1 tPBM effects on MEG/EEG powers observed in sensor space

**Figure 3:**
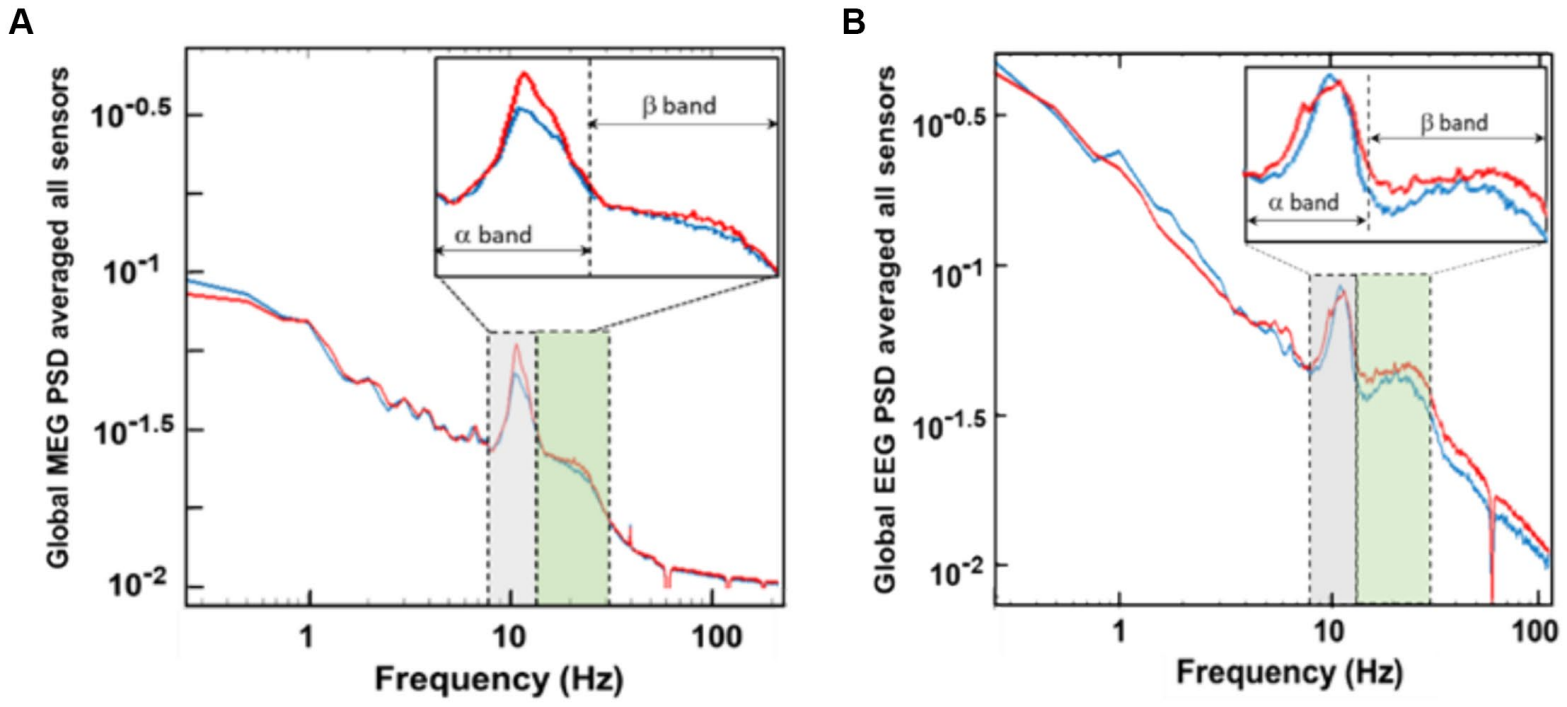
Global PSD curves averaged over **(A)** 204 MEG sensors and **(B)** 64 EEG sensors for 6-min before (blue) and after (red) the 8-min tPBM. This set of MEG/EEG data was obtained concurrently from a randomly selected human participant. The inserts are zoomed PSD curves in the spectral range of the alpha (α: 8–12 Hz) and beta (β: 13–30 Hz) frequency bands.

After data pre-processing, global PSD curves were first calculated for each of the MEG sensors (i.e., 204 planar gradiometers) and then averaged over all the sensors for the 6-min pre-and post-tPBM periods. Figures 3A,B show examples of such global PSD curves from both MEG and EEG measurements, respectively, of a participant during 6-min pre-and post-tPBM epochs.

**Figure 4:**
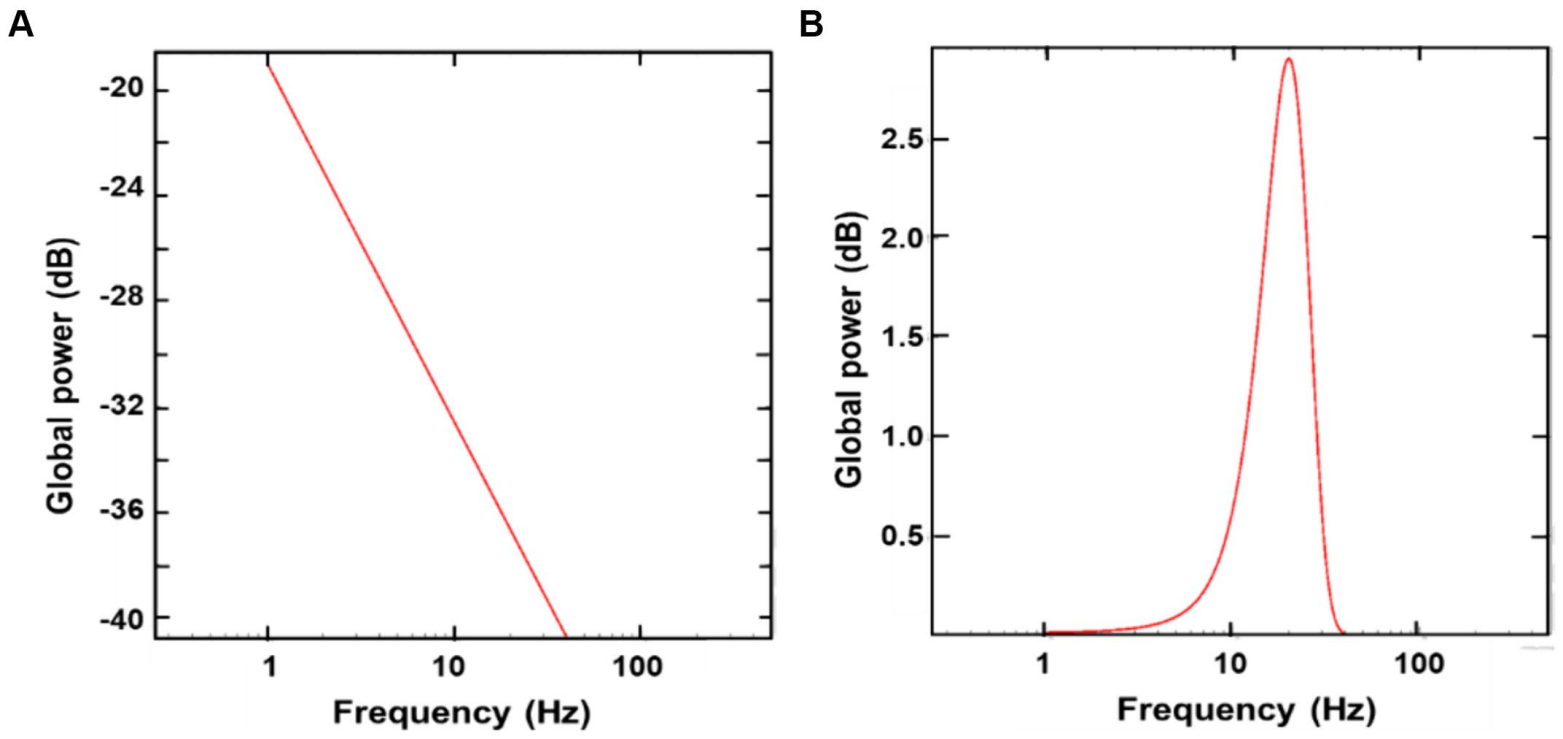
Extracted aperiodic **(A)** and periodic **(B)** features from the combined MEG/EEG PSD averaged across all subjects (*n* = 25), using Global FOOOF analysis. In panel **(A)**, there is a high degree of aperiodic noise in the delta (1–3 Hz) and theta (4–7 Hz) bands; in panel **(B)**, a large spike is shown in periodic activity in the alpha (8–12 Hz) and beta (13–30 Hz) bands.

To accurately attribute these changes in MEG/EEG powers to oscillatory activity affected by tPBM, it is essential to separate aperiodic activity from oscillatory activity. After performing FOOOF analysis on both MEG and EEG data, we were able to split the electrophysiological power spectra into aperiodic and periodic components from each of the MEG/EEG PSD pre-and post-tPBM. For example, Figure 4 shows aperiodic (Figure 4A) and periodic (Figure 4B) features extracted from the combined MEG/EEG PSD in the 0.5–40 Hz region, which is the main frequency band of tPBM-induced significance in our data, averaged across all subjects (*n* = 25). Figure 4A shows a high degree of aperiodic noise in the delta (1–3 Hz) and theta (4–7 Hz) regions, whereas Figure 4B illustrates a large spike in periodic activity in the alpha (8–12 Hz) and beta (13–30 Hz) bands. These results support the significant increase in oscillatory power by tPBM in these two frequency bands.

**Figure 5:**
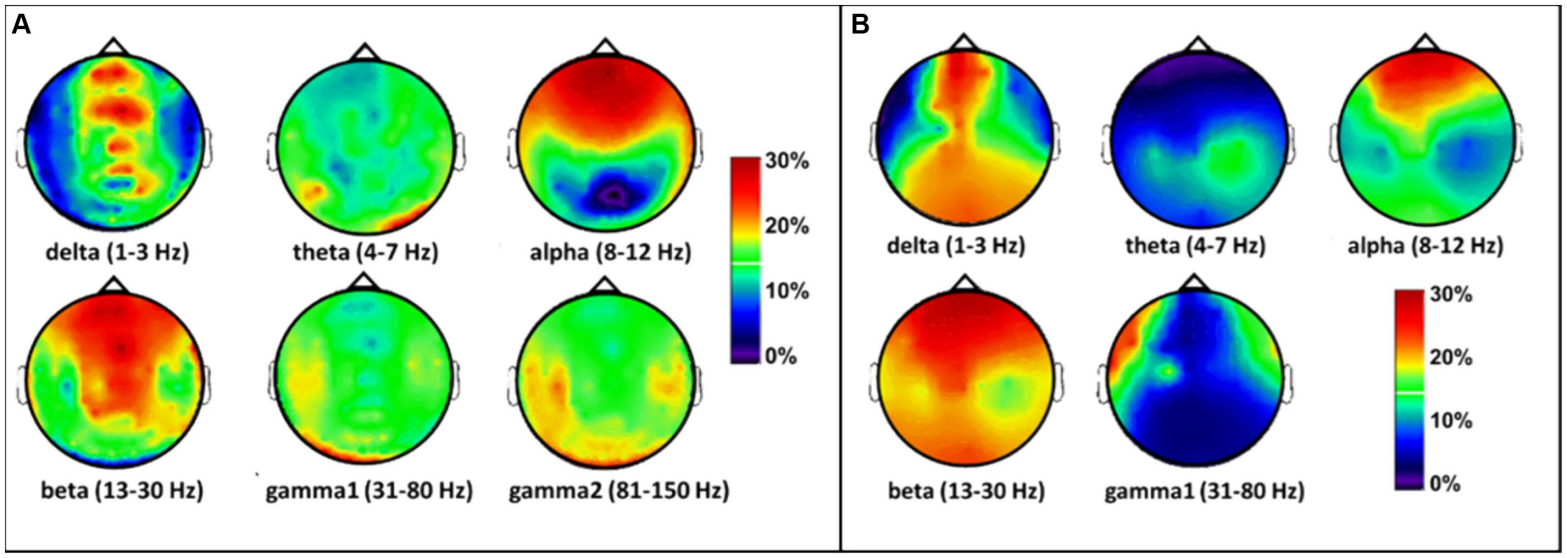
Topographies of tPBM-induced percent/relative alterations (%) with respect to the baseline (pre-tPBM) in **(A)** MEG and **(B)** EEG power in the sensor space in the six frequency bands. The color bar represents the percentage increase in MEG/EEG power with respect to the baseline or the pre-stimulation condition [see Eq. (1)]. Dark blue: relative power increase < 10%; red and dark red: relative power increase > 20%.

Next, to observe the spatial effects of tPBM on the MEG/EEG spectral power in sensor space, we followed Steps 2 and 3 by calculating individual PSD curves for each sensor and determined the band-averaged MEG/EEG powers for all six (or five) bands during pre-or post-tPBM epochs. Specifically, we quantified the percent changes in the MEG/EEG signal power [see Eq. (1)] by computing the tPBM-induced relative PSD power changes for each sensor at each of the respective frequency bands. The sensor-based percentage alterations in MEG power were plotted as interpolated topographies on a standard circular head model (Figure 5A), with the most pronounced increases (>20% with respect to the baseline/pre-tPBM) in the delta, alpha, and beta bands. An increase in power appeared in the frontal to central regions near the site of the tPBM. Following the same data processing steps, we also obtained five interpolated topographies of EEG power percentage alterations in the five frequency bands, as shown in Figure 5B. Note that the tPBM-induced alteration patterns in both MEG and EEG power were highly consistent in both spatial and spectral aspects.

### 3.2 tPBM effects on MEG/EEG powers observed in source space

**Figure 6:**
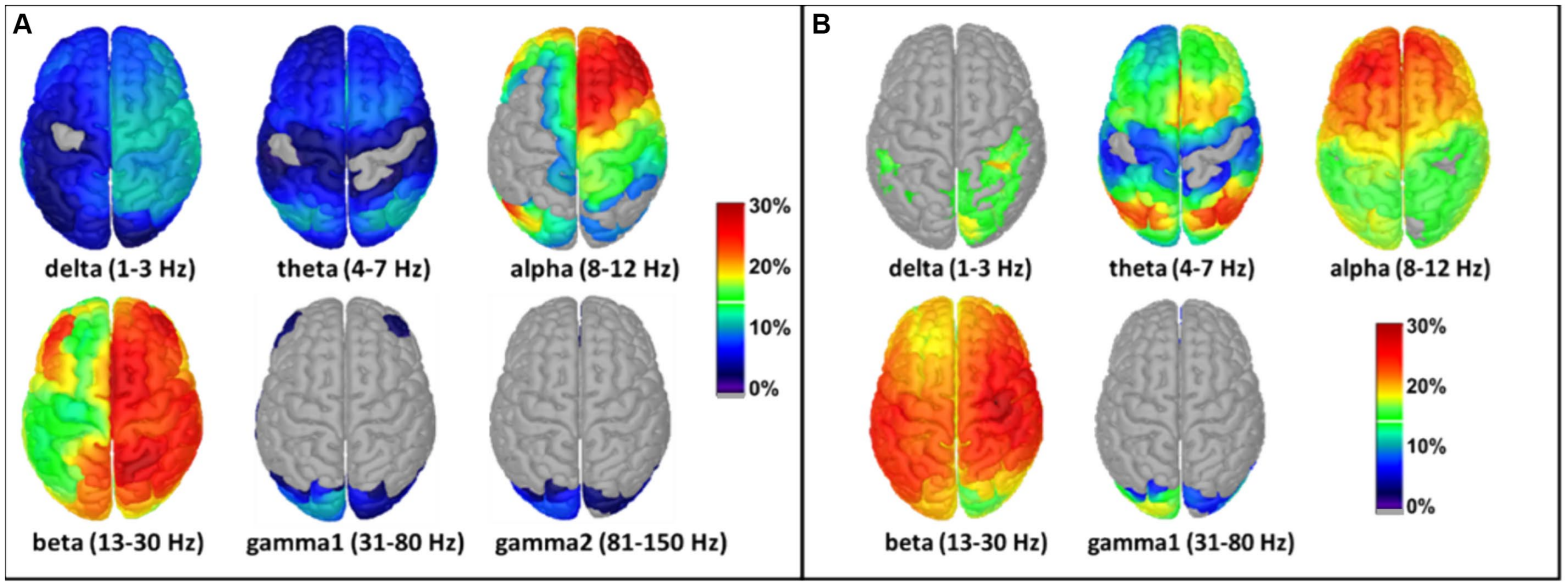
Effects of tPBM on voxel-wise relative PSD power increase (%) obtained using the constrained modeling/solution for **(A)** MEG and **(B)** EEG measurements. The relative PSD changes were calculated by averaging the pre-tPBM and post-tPBM epochs across all subjects, followed by normalized power changes with respect to the pre-tPBM power in each voxel in each of the six/five frequency bands [see Eq. (1)]. Results were thresholded at 1% power changes for ease of viewing. The color bar represents the percentage increase in the MEG/EEG power with respect to pre-tPBM. The stimulation location of the tPBM was near the EEG location of the FP2. Dark blue: relative power increase < 10%; red and dark red: relative power increase > 20%; gray: below a threshold of 1% increase and non-significant change.

The Welch method was also employed to calculate tPBM-induced effects in source space. Constrained modeling was performed after subtracting the pre-tPBM power from the post-tPBM power to generate voxel-wise power differences. Similar to the format of the topographies seen in sensor space (Figure 5), the source imaging solutions of tPBM effects in source space are presented as relative (percentage) changes in MEG/EEG power with respect to the pre-tPBM baseline, as shown in Figure 6. 8-min of tPBM facilitated increases in 6-min-averaged MEG/EEG power mainly in the alpha and beta bands (Figure 6). Specifically, the alpha band demonstrated a 15%–30% increase in power in the frontal, central, and parietal regions on the ipsilateral (right) side (with respect to the stimulation site), while the beta band exhibited a 25%–30% increase in power widespread throughout the ipsilateral cortex. Again, the tPBM-induced MEG and EEG power alterations in source space showed similar trends in spatial (frontal, central, and ipsilateral) and spectral (alpha and beta bands) aspects, while MEG source images showed more ipsilateral effects than the EEG source images.

**Figure 7:**
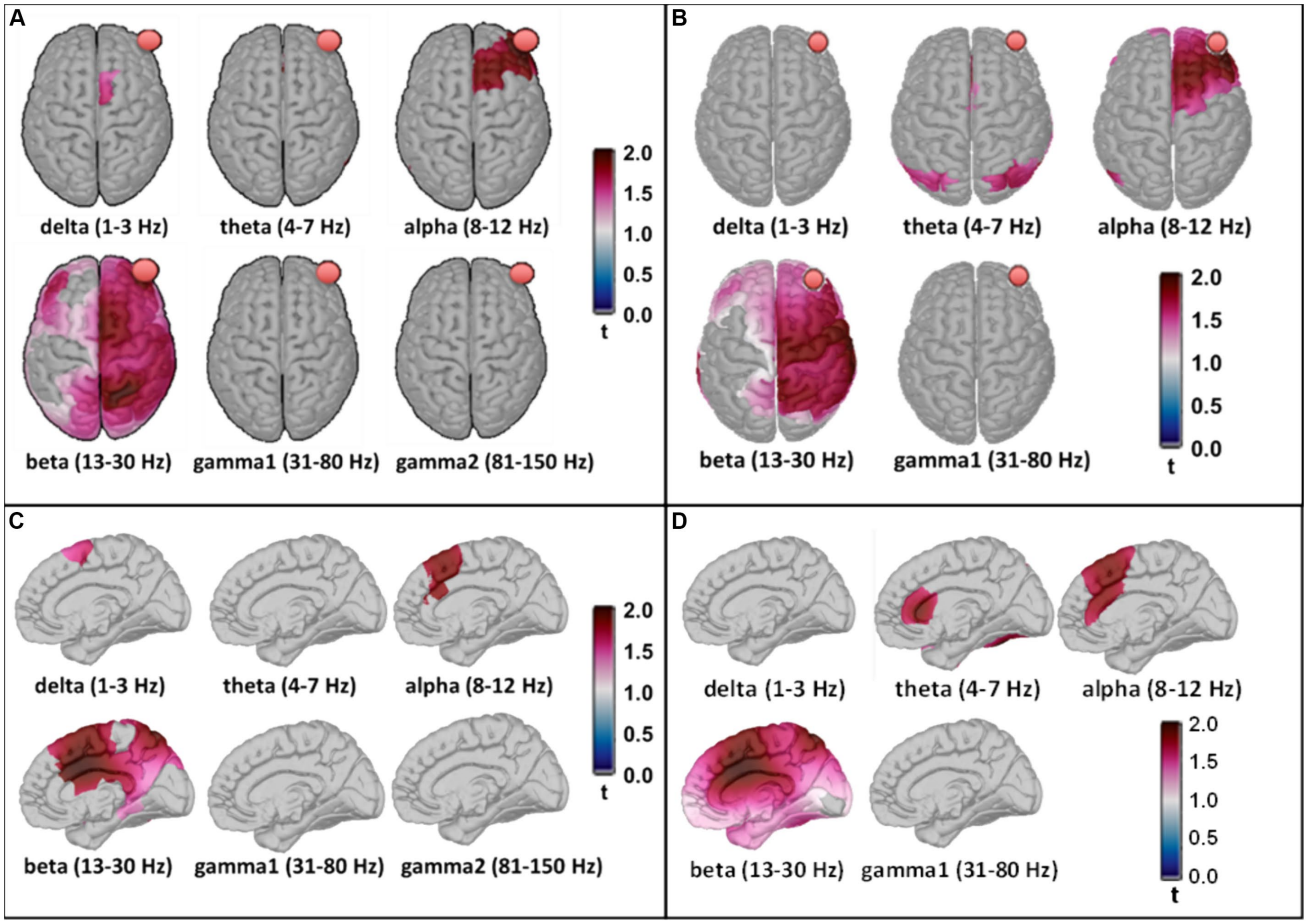
Panels **(A,B)** show statistically significant effects of tPBM on MEG and EEG power, respectively, projected onto the constrained (i.e., on the cortex) source space. The red balls in all panels indicate the location of the tPBM site corresponding to FP2 in the EEG. Panels **(C,D)** present corresponding significant effects of tPBM in a sagittal view of the right hemisphere, respectively, for MEG and EEG. The t-values were scaled based on their magnitude; only those exceeding the statistical threshold of *p* < 0.05 are depicted.

After family wise error correction using cluster-based permutation statistics in Brainstorm, PSD source space maps for both MEG and EEG measurements are presented with cortical views in Figures 7A,B. Significant increases in electrophysiological activity following 8-min tPBM occurred (1) surrounding the site of stimulation in the alpha band and (2) spreading across the ipsilateral hemisphere in the beta band. These two specific observations were highly consistent between the brain/cortex images derived from the concurrently measured MEG and EEG time series.

Moreover, discrepancies seemingly arise between the sensor (Figure 5) and source (Figure 6) space evaluations of tPBM in the MEG. Specifically, relative changes in delta and theta band activities in the sensor topographies with clusters exhibit 10 to 30% changes, whereas the source space activity changes are all limited to below 10%. We expect or assume that these discrepancies might be attributed to the inherent limitations of source space reconstruction techniques, which could underestimate activity in deeper or more complex brain regions. To address this point, we also showed a sagittal view of significant changes in electrophysiological power in both MEG and EEG source space activity in Figures 7C,D, respectively. These two figure panels provide a more comprehensive visualization of the deep spatial distribution of changes in all five frequency band activities. It is clear that right-forehead 8-min tPBM induced significant increases in electrophysiological activity only in the alpha and beta bands with consistent reconstructed sources from both MEG and EEG concurrent measurements, but no significant tPBM-induced changes in delta and theta frequency bands.

## 4 Discussion

There have been numerous human studies *in vivo* supporting the proposed mechanism of action that NIR light facilitates non-invasive photo-oxidation of cytochrome-c-oxidase within the mitochondria of cerebral tissue in both healthy humans and patients with neurological disorders (Hamblin, 2016; Wang et al., 2017; Hamblin and Huang, 2019; Gonzalez-Lima, 2021; Nizamutdinov et al., 2021, 2022; Qu et al., 2022; Zhao et al., 2022). However, few reports have investigated the electrophysiological responses to tPBM (Zomorrodi et al., 2019; Ghaderi et al., 2021; Wang et al., 2021). Prior investigations on this topic were based on measurements of 32-or 64-channel EEG only, which has limited spatial resolution for cortical neuroimaging. This information is critical for understanding tPBM-induced neuro-electrophysiological effects in the human brain. Here, we used concurrent MEG and EEG as advanced neuroimaging tools to support and validate previous EEG findings, while improving on the spatial resolution of cortical electrophysiological images in response to tPBM using independent electrophysiologic methods.

### 4.1 Consistent MEG and EEG power increases in alpha and beta bands

As shown in Figures 5–7, tPBM has a significant post-stimulation impact on the power of electrophysiological oscillations in the alpha (8–12 Hz) and beta (13–30 Hz) bands, in both sensor and source space derived from both MEG and EEG measurements. The MEG source images, resulted from 204 gradiometers, should enable a higher spatial resolution than that obtained from the concurrent 64-channel EEG measurements. After cluster-based permutation statistical analysis, both MEG and EEG brain source images in response to 8-min tPBM were in excellent agreement in that tPBM significantly increased (1) the alpha power near the right prefrontal or frontal cortex, and (2) the beta power in the ipsilateral hemisphere (on the same side of tPBM) spreading from the frontal to central and then to parietal cortical regions. 8-min of prefrontal 1,064-nm laser tPBM facilitates significant enhancement in electrophysiological activity in both alpha and beta oscillations.

The alterations in alpha and beta frequency power in the cortex are of significant interest as recent studies have documented their prevalence in several resting-state studies (Rosanova et al., 2009; Barry et al., 2019; Capilla et al., 2022). These frequencies have also been recognized in previous research as playing a critical role in various auditory (Teplan et al., 2006a,b; Kumar et al., 2016) and visual (Wróbel, 2000) data processing pathways within the brain. However, the exact connection or association between these frequency bands and tPBM stimulation is unclear and needs further investigation. Future task-based studies, specifically designed to elicit a response in these frequency bands, are necessary to fully elucidate the relationship between tPBM and the alterations in alpha and beta power within the brain.

### 4.2 Localizations and significance of tPBM effects

Source localization in the prefrontal cortex (Figure 7) exhibited significant increases in MEG/EEG alpha power after 8 min of tPBM. The sources were located more on the ipsilateral side and extended from the right prefrontal (near or at the light delivery site), right dorsolateral prefrontal, to right medial prefrontal regions. The latter two regions are part of the default mode network. Furthermore, significant increases in MEG/EEG beta power occurred in the cortical regions, both spread over the ipsilateral hemisphere and spanned the contralateral areas in a reduced proportion. Notably, these beta-power-stimulated regions include the bilateral dorsolateral prefrontal region, which is a crucial cortical area for complex cognitive activities, such as planning and decision-making; the right parietal cortex, which is involved in spatial awareness and sensory integration; and both the right and left occipital regions, associated with visual processing (Culham and Kanwisher, 2001; Curtis and D'Esposito, 2003; Bullmore and Sporns, 2009; Bressler and Menon, 2010; Levy and Wagner, 2011; Huang and Sereno, 2013). The involvement of these regions suggests a widespread activation pattern with potential network engagement. This could contribute to the observed effects of tPBM on improved cognition and behavior reported in other studies. This set of beta source localizations indicates a more generalized effect associated with tPBM stimulation, with the most significant effect still at the lateral site of tPBM stimulation.

All tPBM-stimulated cerebral regions found in this study were highly consistent with those reported using an EEG-derived network analysis based on the group singular value decomposition algorithm and the eLORETA software package (Wang et al., 2022). The consistency of the results obtained using the independent modalities from two different groups of participants and operators provided strong evidence of the reliability of the measurements. Moreover, the observation that tPBM significantly stimulated more widespread cerebral regions at the beta rhythm provides a rationale for why only in the beta frequency band, tPBM was shown to enhance the complexity of the global brain network, augment local information flow, and integrate oscillations across prefrontal cortical regions (Shahdadian et al., 2022). Overall, our MEG and EEG findings contribute to a growing body of evidence that tPBM facilitates significant alterations in neuro-electrophysiological activity (Zomorrodi et al., 2019).

It is well known that specific regions of the cortex, such as the right frontal, parietal, and occipital regions, are cerebral sources for human cognitive functions (Sehatpour et al., 2008; Tse et al., 2021). This study using MEG and EEG neuroimaging revealed significant alterations in alpha and beta power following an 8-min tPBM intervention across similar regions. Moreover, numerous studies have reported that tPBM facilitates the improvement of cognitive function and reduces the symptoms of neurodegenerative diseases (Rojas and Gonzalez-Lima, 2013; Purushothuman et al., 2014; Cassano et al., 2015; Farfara et al., 2015; Blanco et al., 2017). tPBM may be a promising therapeutic tool for treating certain neurological diseases.

### 4.3 Consistent EEG power alterations between this study and previous reports

The results shown in Figure 5 correspond to tPBM-induced percentage changes of EEG alpha and beta power in sensor space with results similar to previous EEG studies (Wang et al., 2019; Zomorrodi et al., 2019; Wang et al., 2021). The same experimental and tPBM protocols were used in those studies with differences in post-tPBM of 6 min (current study) vs. 3 min (prior studies; Wang et al., 2019, 2021). Regardless of the temporal length difference in post-tPBM data acquisition, the EEG alpha power topographies between these studies are highly consistent. The observation that post-tPBM increases in beta power in the previous study were weaker than those in this study (Figure 5) can be attributed to a shorter data acquisition time (3 vs. 6 min, respectively). Overall, the tPBM-induced EEG power alterations in both the alpha and beta bands in sensor space were in good agreement between this study and previous reports. In addition, our results highlight significant post-tPBM effects lasting for at least 6 min after stimulation. The duration of significant tPBM effects however is not known.

### 4.4 Possible thermal impacts on the MEG/EEG results

Because tPBM uses light and thus may create non-negligible thermal effects, especially with a 1,064-nm laser, it is reasonable to question whether the observed electrophysiological signal changes resulted from tissue heating by the laser. However, a recent study of Dmochowski et al. (2020) employed magnetic resonance thermometry to measure brain temperature during 10-min tPBM (*n* = 20) with an 808-nm laser and found no significant temperature differences in the cortex between active and sham stimulation. Another group conducted computer simulations of the motor cortex tPBM at 500 mW/cm^2^ at three wavelengths (630, 700, and 810 nm). They found a temperature increase in the scalp below 0.25°C and a minimal temperature increase in the gray matter of less than 0.04°C at 810 nm. Similar tPBM heating outcomes were obtained for 630 nm and 700 nm light (Bhattacharya and Dutta, 2019). More specifically, one of our recent studies demonstrated that 1,064-nm tPBM and thermal stimulations induced significantly different topographies of changes in EEG alpha and beta power, providing evidence to support that laser-induced heat on the human forehead is not a mechanistic source causing increases in EEG power during and after tPBM (Wang et al., 2021). Accordingly, the observed changes in MEG spectral powers are unlikely to result from thermal effects.

### 4.5 Several technical aspects learned from this study

We acknowledge that the inclusion of data analysis from the corresponding sham-conditioned measurements would be ideal to overcome potential confounding factors. However, our current data analysis took a simpler statistical approach to compare the respective MEG/EEG parameters pre-and post-intervention to assess the impacts of tPBM. More comprehensive statistical analyses should be performed in future studies.

Given the current analysis methodology for MEG/EEG data, a key concern was the specific confounding effect of short-time sleepiness spent within the MEG scanner, which would affect changes in alpha-band activity related to vigilance. To address this concern, it is imperative to consider both the context of our study and relevant findings in the literature. Gibbings et al. (2021) investigated the EEG and behavioral correlates of mild sleep deprivation, a condition analogous to the potential effects of short-term drowsiness on vigilance. They reported that vigilance decrement or drowsiness is typically associated with an increase in alpha band power in the occipital region and a general decrease in beta band power, which are indicative of reduced alertness or sleepiness. In contrast, our observations showed neither the anticipated increase in occipital alpha power nor the expected decrease in beta band power. Instead, the electrophysiological data we collected were indicative of maintained, if not enhanced, cognitive engagement and alertness, inconsistent with the patterns of decreased vigilance described by Gibbings et al. (2021). There is also a larger body of studies with similar supporting evidence, specifically in the alpha and beta frequency bands (Lockley et al., 2006; van Dijk et al., 2008; Lew et al., 2021). This discrepancy between the context of our study and relevant findings in the literature underscores our position that the approximately 20-min resting state in the MEG scanner environment in our study does not significantly compromise alpha or beta frequency band data.

We did not consider the tPBM effects caused by differences in skin pigmentation in this study. In theory, darker or lighter skin pigmentation can affect the absorption of light used for tPBM.

Last, head movements are a problem in EEG data collection. While the EEG coordinate system is always aligned to the head, as it is fixed to the scalp, EEG data must always be scrutinized for large muscle movement artifacts. However, use of the HPI coil in our MEG scanning effectively tracked motions and made corrections using the MEGIN MAXFilter program (Taulu and Simola, 2006; Medvedovsky et al., 2007).

### 4.6 Comparison to other neuromodulation technologies

tPBM, TMS, and tDCS are noninvasive neurostimulation methods with diverse mechanisms of action and neurophysiological effects. While TMS uses magnetic fields to induce electric currents in the neurons of the brain (Aberra et al., 2020) and tDCS employs direct electrical currents to modulate neuronal activity (Stagg et al., 2018), tPBM utilizes near-infrared light to affect mitochondrial activity and cellular metabolism, potentially leading to alterations in neural activity. Our findings demonstrated significant changes in alpha and beta band activities following tPBM, suggesting its efficacy in modulating cortical excitability and neural dynamics. This is particularly relevant in the context of cognitive enhancement and mental health applications, where the modulation of specific frequency bands has been linked to therapeutic outcomes. Comparatively, TMS and tDCS have been shown to influence cortical excitability with varying degrees of specificity and penetration depth, often depending on the parameters used (Ardolino et al., 2005; Zaghi et al., 2010). As suggested by this study, the distinct advantage of tPBM lies in its ability to induce widespread neurophysiological changes without direct electrical stimulation, potentially reducing discomfort and increasing the feasibility of its application.

### 4.7 Limitations and future work

This study has several limitations. (1) Although MEG can identify electrophysiological activity, it has limitations in measuring deeper brain effects of tPBM. This is because of the magnetic nature of the MEG device, which detects magnetic fields orders of magnitude smaller than those detected by EEG (Malmivuo, 2012). This limitation becomes increasingly prevalent as the signal amplitude decreases with time after stimulation. (2) The sensors used in MEG preferentially detect magnetic fields perpendicular to the orientation of the sensor surfaces (Srinivasan et al., 2006; Ahlfors et al., 2010). Thus, MEG is not as sensitive to tPBM-induced magnetic signals parallel to the sensor surface. On the other hand, EEG can detect electromagnetic fields perpendicular and parallel to sensor surfaces. Thus, an advanced algorithm that jointly analyzes both EEG and MEG signals may be advantageous in providing a more comprehensive assessment of tPBM with better spatial and temporal resolution. (3) We also acknowledge the limitation associated with not employing a neuronavigation system to precisely identify the stimulation area, which is commonly used in TMS studies. This could potentially introduce variability in our findings due to the less precise localization of the stimulated area. We recognize that neuronavigation could enhance the specificity of our results by ensuring consistent targeting across participants. However, the observed widespread effects of tPBM suggest that tPBM affects broader neural networks, which may mitigate some concerns regarding the exact stimulation site. Future studies could benefit from incorporating neuronavigation to further explore the specificity of the tPBM effects. (4) Last, we recognize the limitation of using the identity matrix for applying noise covariance in source modeling, as opposed to deriving it from empty room noise recordings. While our approach was practical and aligned with common practices in the field, we understand that it may oversimplify the assumption that noise is uncorrelated across sensors. This could potentially impact the accuracy of the source localization and noise estimation. Thus, future research should consider employing comprehensive methods for noise covariance estimation, such as using empty room recordings, to improve the reliability of source modeling results.

## 5 Conclusion

In this study, we report the first simultaneous MEG and EEG source imaging of electrophysiological activity in response to acute 8-min 1,064-nm right prefrontal tPBM. Our results obtained from 25 healthy participants indicated that the 8-min tPBM enabled significant increases in both the alpha (8–12 Hz) and beta (13–30 Hz) frequency power across multiple cortical regions. This observation was confirmed by both MEG and EEG source modeling (corrected *p* < 0.05). Our findings have important implications in the field of tPBM neuromodulation. Specifically, tPBM-induced oscillatory modulations are not only located near the site of tPBM but also in remote cerebral regions, including the frontal, parietal, and occipital regions, and are more weighted toward the ipsilateral side. Furthermore, the effects persisted for minutes after stimulation. The findings for EEG power alterations are also consistent with those of recent reports and contribute to the growing body of literature on the impact of tPBM on oscillatory activity in the human brain. However, further research is needed to understand the effects of tPBM on brain networks and the longitudinal tPBM effects. In conclusion, this study presents MEG/EEG evidence of electrophysiological effects in cortical regions induced by 8-min right-forehead tPBM and highlights the need for further research in this field.

## Data availability statement

The raw data supporting the conclusions of this article will be made available by the authors upon request, without unduereservation.

## Ethics statement

The studies involving humans were approved by the Institutional Review Board of the University of Texas Southwestern Medical Center at Dallas, Dallas, TX, United States. The studies were conducted in accordance with the local legislation and institutional requirements. The participants provided their written informed consent to participate in this study.

## Author contributions

TP: Investigation, Methodology, Visualization, Data curation, Formal analysis, Software, Writing – original draft. ED: Data curation, Formal analysis, Investigation, Methodology, Software, Validation, Writing – review & editing. AP: Investigation, Methodology, Writing – review & editing, Data curation, Software. JM: Investigation, Methodology, Conceptualization, Project administration, Resources, Supervision, Validation, Writing – review & editing. HL: Conceptualization, Funding acquisition, Investigation, Methodology, Project administration, Resources, Supervision, Visualization, Writing – review & editing.
